# Electrical Characterization of Proposed Transpositional Acupoints on the Urinary Bladder Meridian in a Rat Model

**DOI:** 10.1155/2011/295475

**Published:** 2011-02-17

**Authors:** Hyun-Jung Han, Sang-Jun Park, Kwang-sup Soh, Hyoun-Seok Myoung, Kyoung-Joung Lee, Vyacheslav Ogay, Yong-Heum Lee

**Affiliations:** ^1^Biomedical Physics Laboratory, Department of Physics and Astronomy and Center for Theoretical Physics, Seoul National University, Seoul 151-747, Republic of Korea; ^2^Department of Biomedical Engineering, Yonsei University, 234 Maeji-Ri, Heungup-Myeon, Wonju-Si, Gangwon Do 220-710, Republic of Korea; ^3^Lab of Immunochemistry and Immunobiotechnology, National Center for Biotechnology, Astana 010000, Kazakhstan

## Abstract

Specific electrical characteristicsof acupointswere investigated on the urinary bladder (BL) meridian in 14 rats. BL acupointsand non-acupoints on the back were selected and their electrical voltages were measured by using aSPACsystem.The mean voltages of each point or each line were statistically analyzed by using the ANOVA test.The BL meridian showed voltages higher than those of the reference line (*P* < .05). Bilateral 1st BL lines presented higher voltages than bilateral 2nd BL lines (*P* < .05). Most BL acupoints had voltageshigher than those for the corresponding reference points (*P* < .05). In particular, theright BL16 exhibited the biggest difference from the reference point, followed by the left extra BL point-2, the right BL27, the left BL17, and theleft BL45. Additionally, the distributions of neurofilamentsfor several points were investigated by using immunohistochemistry. There was a trend for the BL acupoints to have larger numbers of neurofilaments than the reference points, and that trend seemed to be directly proportional to the difference in voltage between the points.In conclusion, BL acupoints on the back in ratsexhibited specific electric and histologic characteristics. Therefore, those acupointsmay be utilized to investigate the efficacy of acupuncturewith laboratory animals.

## 1. Introduction

Animal studies are a prerequisite for evaluating the efficacy and the mechanism of acupuncture. Therefore, much research on acupuncture has been done using laboratory animals. However, acupoints in laboratory animals, especially rats and mice, have not yet been clearly defined, and the same acupoint may be interpreted differently by different researchers.

Until recently, acupoints in laboratory studies have been selected based on a transpositional method that located the veterinary acupoints by transposing human acupuncture points onto an animal's anatomy [1–4]. Because the anatomy of rats was considered to be nearly identical to that of humans, the locations of the acupoints and the travel routes of the meridians in rats should resemble those in humans [5]. The method has generally been accepted by researchers; in particular, transpositional acupoints for rats and mice were reported by Koh [2, 3]. 

However, the selection of acupoints by using transpositional method differs with the researcher [4]. For example, there was some discrepancy defining the location of the ST 36 point (stomach meridian 36); Kim suggested its location as 5 mm lower and lateral to the anterior tubercle of the tibia [6, 7], and Lim suggested it as 2 mm lateral to the anterior tubercle of the tibia [8]. There are some reasons for these differences. First, the locations of the acupoints may be subjectively interpreted by a researcher because the locations of most acupoints are examined by using a relative anatomical proportion, rather than an absolute number. Second, some anatomical differences exist between animals and humans and even between the species of an animal, so some established acupoints of humans may not correspond directly to those of animals. Such a discrepancy leads to different outcomes even when the same acupoints are used in the laboratory animals. Therefore, the results of acupuncture studies using animals are still controversial, and a serious limitation on analyzing the efficacies of specific acupoints exists.

To solve the above problem and to assist in confirming acupoints, researchers have investigated the characteristics of acupoints, such as the electrical characteristics [9] or excitable muscle/skin-nerve complexes with enriched nerve endings [5]. In particular, the electrical characteristics of acupoints have been widely investigated and are regarded as the most reliable characteristics [10]. Acupoints are generally known to possess increased conductance, and compared to nonacupoints, acupuncture meridians have been proposed to act as conduits for electrical currents [10–22]. 

However, some limitations exist due to the poor quality of some studies: small sample sizes, poor procedural description, lack of rigorous statistical analyses, and limited region for evaluation, such as the extremities [10, 23]. Therefore, further studies are needed to investigate the acupoints and the meridiansat more various sites on the body more exactly.

In this study, we tried to determine several acupoints in a rat by using transpositional acupoints and electric characteristics. Of the large number of acupoints. The acupoints of urinary bladder (BL) meridian on the back, which have frequently been used clinically and experimentally, were investigated in this study. The purpose of this study was to use electrical characteristics to establish the BL acupoints in a rat. 

## 2. Materials and Methods

### 2.1. Animal Preparation

Fourteen Sprague-Dawley rats were utilized in this study. The animals were 8 weeks old, were all female, and weighed about 200 g (200.85 ± 3.20 g). The animals were housed in a temperature-controlled environment (23°C) with 60% relative humidity and a 12-h light/dark cycle. They were anesthetized through i.p. injection of a mixture of ketamine hydrochloride (50 mL/kg) and xylazine hydrochloride (5 mL/kg). The animals were placed in a sternal recumbency, and hair on the back region was completely removed with great care to avoid iatrogenic damage to the skin on the backs. The procedures used in the animal experiment were in full compliance with current international laws and policies (European Convention for the Protection of Vertebrate Animals Used for Experimental and Other Scientific Purposes). The protocols used in this study were approved by the Institute of Laboratory Animal Resources of Seoul National University (SNU-08310-1). 

### 2.2. Selection of the Investigated Points

**Figure 1:**
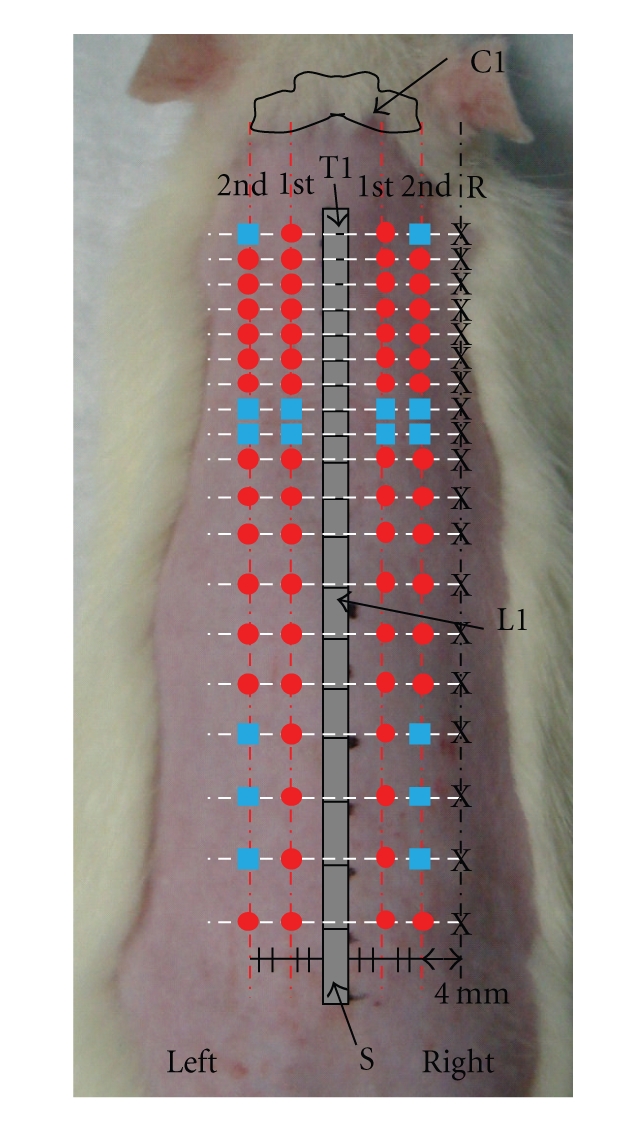
Points investigated to determine the electrical characteristics on the back in a rat. The points include transpositional acupoints of the BL meridian on the back (red circles) and nonacupoints even though those were located on the path of the BL meridian on the back (blue squares). The electrical voltages were compared to values for corresponding reference points (X). C1: atlas, T1: 1st thoracic vertebra, L1: 1st lumbar vertebra, S: sacrum, Gray bar: vertebra from T1 to S (midline of the back), 1st (1st BL line, the longitudinal line extending from the middle point between the 2nd BL line and the midline of the back), 2nd (2nd BL line, the longitudinal line extending from the end of the atlas wing), and R (the line of reference points, 4 mm to the right of the 2nd BL line).

The BL meridian on the back includes two bilateral lines. The first BL line is the inner line, and the second line is the outer line. The selection of BL lines was based on the veterinary acupoints of a dog suggested by Kim et al., for which the 2nd BL line is the longitudinal line extending from the end of the atlas wing, and the 1st BL line is the longitudinal line extending from the middle point between the 2nd BL line and the midline of the back [24]. Each acupoint of the 1st and the 2nd BL lines was selected lateral to the caudal border of the spinous process of each vertebra and ranged from the 1st thoracic vertebrae (T1) to the 6th lumbar vertebrae (L6). The nomenclature used for the individual acupoints in this study follows the nomenclature for the traditional acupoints corresponding to the locations (Table 1). Although 8 points on the path of the BL meridian, such as the points related to the 8th and the 9th thoracic vertebrae on the first BL line and to the 1st, 8th, and 9th thoracic and the 3rd, 4th, and 5th lumbar vertebrae on the second BL line were not previously established acupoints, they were included. The nomenclatures for these points are represented by EBL—(extra BL acupoints) in this study and ranged from EBL-1 to EBL-8 (Table 1). As a reference point, a non-acupoint located laterally 4 mm from the right 2nd BL line and located on the same level as the individual BL acupoint was investigated. A diagram of the investigated points is shown in Figure 1. 

### 2.3. Discrimination of Acupoints by Using the SPAC System

**Figure 2:**
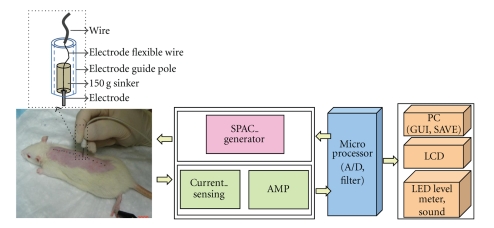
Block diagram of the SPAC system. The black dotted rectangular area indicates of the electrode in the SPAC system. An electrode guide pole encloses the electrode which is linked with a 150-g sinker.

The electrical voltage of each point was measured three times by using a (SPAC single power alternating current) system. The performance of the system is divided into three functions—generating the SPAC stimulus pattern, measuring and displaying the current value, and transmitting data to a computer. The system was designed with a Microchip PIC18f252 microprocessor. The waveform generation circuit generates the SPAC stimulus pattern.  Figure 2 presents a block diagram and a schematic the system.

At first, the microprocessor outputs a pulse of 4 kHz at 5 V. The SPAC system supplies current to the body. The supplied current is converted to a voltage. Next, the voltage is transferred to a personal computer. Then, a raw data point, which is the impedance value of the detected acupoint, is displayed on a LED level-meter and is indicated by a sound. The data transferred to the PC are displayed on an LCD by using a GUI program (LabVIEW). 

### 2.4. Immunohistochemistry

To compare the distributions of neurofilaments between BL acupoints and reference points, we excised tissue explants (~1 cm^2^), including skin and muscle (3 mm × 3 mm width), from the measured points of rats and fixed them overnight at 4°C in a fixative consisting of 0.5% paraformaldehyde (Sigma, USA) and 15% (v/v) saturated picric acid (Sigma, USA) in a 0.1 M sodium phosphate buffer (SPB, pH 7.0) [25]. Prior to sectioning, the tissue samples were infiltrated in O.C.T. compound (Sakura Finetek, USA) and frozen in isopentane at the temperature of liquid nitrogen. The frozen samples were sectioned in the horizontal plane by using a cryptome (Microm Lab. HM 505E, Germany) at 100 *μ*m. Tissue sections were rinsed in PBS for 15 min to remove the O.C.T. embedding material and were incubated in a 3% sodium-deoxycholate solution (Fluka, Italy) for 4 h at room temperature under mild agitation in a 24-well plate (Falcon, USA). The specimens were rinsed twice with distilled water and then three times with PBS for 1 h each. The sections were incubated in 10% normal goat serum (NGS) (Chemicon Inc., USA) overnight at 4°C and then for 1 day at 4°C in rabbit anti-neurofilament 160 kD polyclonal antibody (1 : 1000, Abcam, UK). After the sections had been washed with PBS, they were incubated in Alexa Flour 488 goat anti-rabbit IgG (H+L) (1 : 500) (Invitrogen, USA) for 1 day at 4°C. All antibodies were diluted in 10% NGS containing 0.1% NaN3 (pH 7.4). After each incubation with antibodies, sections were washed in three times with PBS. 

Tissue sections were stained with DNA-specific dye (DAPI), mounted in antifading medium, and observed with a stereomicroscope (MVX-10, Olympus, Japan). Images of thick sections were obtained by using a digital camera (DP-70, Olympus, Japan). Ten serial images from each sample were adjusted, merged into a common image, and analyzed with computer software (Image J, v. 1.37, USA). 

### 2.5. Statistical Analysis

The voltage of each measure point was represented by an average (mean), standard deviation (SD), and standard error. The ANOVA test was applied to compare the mean voltages of the measure points or to compare each BL to a reference line. Then, Tukey's multiple comparison test was performed for multiple comparisons; a value of *P* < .05 was considered significant. 

## 3. Results

### 3.1. Mean Voltages of the Acupoints on the BL Meridian and Reference Points

**Figure 3:**
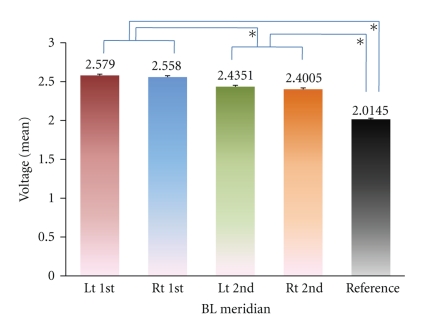
Mean voltage for each BL meridian on the back in a rat model. All four lines belonging to the BL meridian represent significantly higher mean voltages than the mean values for the reference points (nonacupoints on the back). In particular, bilateral 1st BL lines show significantly higher voltages than bilateral 2nd BL lines *P = .000).

The mean voltages of the acupoints on the bilateral 1st and 2nd BL lines were significantly higher than those of the reference points (Figure 3). The mean (±SD) voltages were 2.579 (±0.482) for the left 1st BL line, 2.558 (±0.514) for the right 1st BL, 2.435 (±0.515) for the left 2nd BL, and 2.401 (±0.514) for the right 2nd BL meridian. The mean voltage of the reference points was 2.015 (±0.389). The mean voltage of all BL meridians was significantly higher than the mean voltage of all reference points (*P* < .05). In particular, the bilateral 1st BL lines exhibited a significantly higher voltage than the bilateral 2nd BL lines (*P* < .05). 

**Figure 4:**
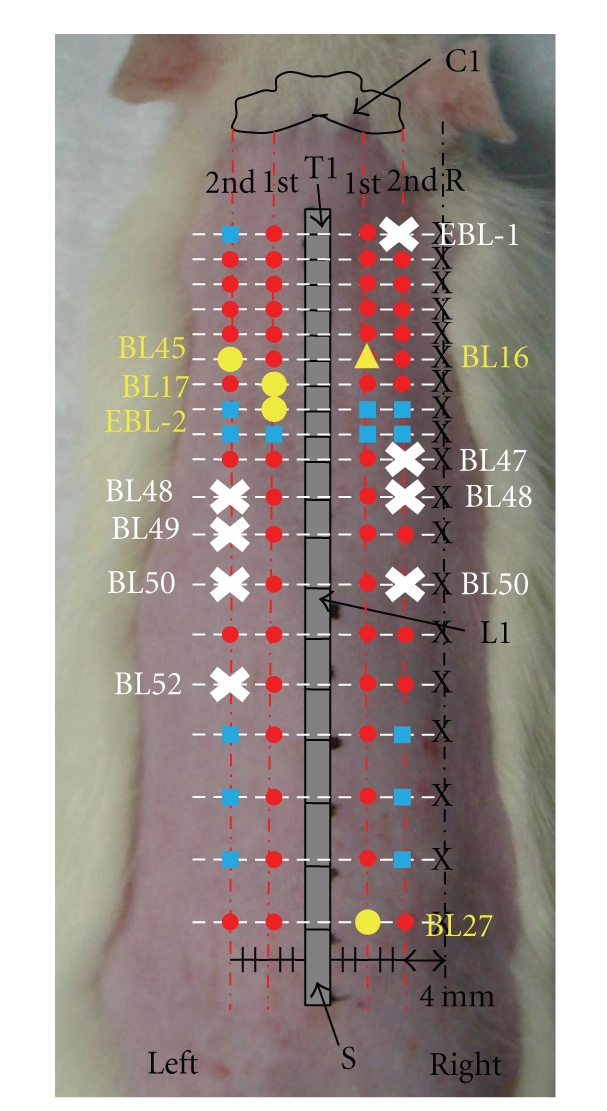
Most of the points on the BL meridian (red circles and blue squares) had significantly higher mean voltages than the corresponding reference points (black X; *P* < .05). The difference inmean voltages between the BL acupoint and the corresponding reference point, Rt BL16, was the biggest difference (yellow triangle), followed by Lt EBL-2, Rt BL27, Lt BL17, and Lt BL45 (yellow circles). However, the mean voltages for several points on the 2nd BL line, including Rt EBL-1, BL 47, BL 48, BL 50 and Lt BL 48, BL 49, BL 50, BL 52 (white X), were not significantly different from the values for the corresponding reference points.

In the comparison of the mean voltages between the individual pointson the same transverse line extending from the vertebrae, most BL acupoints had significantly higher voltages than the corresponding reference points (*P* < .05). All acupoints on the bilateral1st BL lines had significantly higher mean voltages than the corresponding reference points. On the other hand, the mean voltages of several points on the bilateral 2nd lines were not significantly different from those for the corresponding reference points: right EBL-1, BL47, BL48, BL 50 and left BL48, BL49, BL 50, BL 52 (Figure 4).

**Figure 5:**
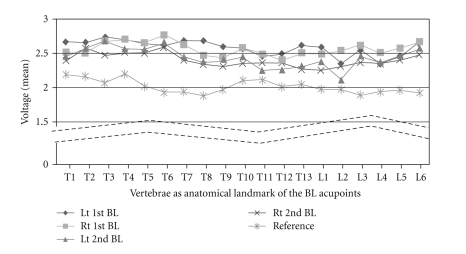
Mean voltage for each BL acupoint on the back in a rat model. The values for the BL acupoints ranged from 2.1124 (Lt BL52) to 2.761 (Rt BL16) while the values for the corresponding reference points ranged from 1.879 (level with T8) to 2.1969 (level with T4). The biggest difference in the mean voltages between a BL acupoint and a corresponding reference point was 0.8386 for Rt BL16 (level with T6) while the lowest value was 0.1321 for Lt BL 52 (level with L2).

The mean voltages of the BL acupoints ranged from 2.1124 to 2.761 (Figure 5). The highest mean voltage was for Rt BL16 (2.761), followed by Lt BL13 (2.7336), Lt BL14 (2.7005), Rt BL14 (2.6933), and Lt BL17 (2.6807). The lowest mean voltage was 2.1124 for Lt BL52. The mean voltages of the corresponding reference points ranged from 1.879 (transverse line extending from T8) to 2.1969 (transverse line extending from T4). As for the differences in mean voltages between the BL acupoints and the corresponding reference points, Rt BL16exhibited the biggest difference (0.8386), followed by Lt EBL-2 (0.7836), Rt BL27 (0.7357), Lt BL17 (0.7286), and Lt BL45 (0.7286), while Lt BL52 showed the smallest difference (0.1321) (Figures 4 and 5). 

### 3.2. Distribution of Neurofilaments in BL Acupoints and Reference Points

**Figure 6:**
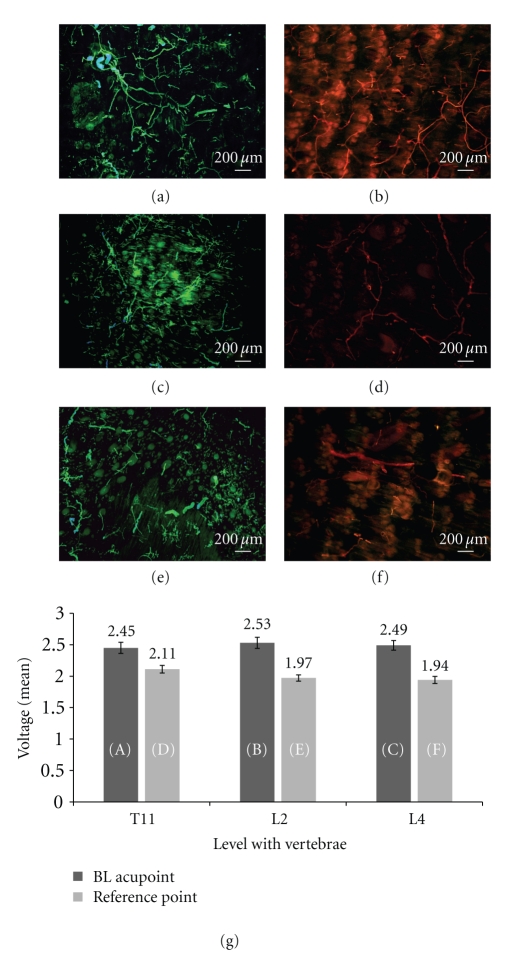
Distribution of neurofilaments between BL acupoints (a, b, c) and the corresponding reference points (d, e, f): (a) and (d) left BL 19 and its corresponding reference point, (b) and (e) right BL23 and its corresponding reference point, and (c) and (f) right BL25 and its corresponding reference point. (g) Comparison of the mean voltages between the BL acupoints (a, b, c) and the reference points (d, e, f). (a) and (d) show the least differences in mean voltages.

To compare the distributions of neurofilaments between BL acupoints and reference points, we selected several BL acupoints for immunohistochemistry. The acupoints were compared with the corresponding reference points to eliminateregional differencesin the distributions of neurofilaments. As a result, a distinct trend, most BL acupoints had larger numbers of neurofilaments than the corresponding reference points (Figure 6), was noted. The difference in the distributions of neurofilaments between BL acupoints and reference points was related to the difference in mean voltages between the points. BL acupoints and reference points exhibiting little difference in the mean voltages seemed to exhibit little difference in the distributions of neurofilaments (Figures 6(a) and 6(d)). On the other hand, points having a big difference in the mean voltages seemed to also have a big difference in the distributions of neurofilaments (Figures 6(b) and 6(e), and 6(c), and 6(f)). 

## 4. Discussion

To investigate the electrical characteristics of transpositinal acupoints, we selected the BL meridian, especially on the back, for two reasons: its significant usefulness in clinical and experimental study and its ease of access. The BL meridian has significant meanings for experiments on and to clinics of Oriental medicine. In particular, a well-known concept in Oriental medicine is that most acupoints of the BL meridian on the back are organ-associated points; that a certain acupoint in a region reflects the condition of a certain organ, for example, BL13 is a lung-associated point and BL23 is a kidney-associated point, and so forth [24, 26]. The points have been named *Back-shu points* and can be used in Oriental medicineto diagnose and treat simultaneously disorders of the related organ. 

The acupoints of the BL meridian on the back are easy to access due to obvious anatomical landmarks such as vertebra. However, according to previous studies, the selections of the 1st and the 2nd BL lines by using the transpositional method are a little different. In human medicine, the 1st and the 2nd BL lines are 1.5 and 3 *cun* lateral to the midline of the back. However, the proportional method (using a concept of *cun*) for human acupoint location is too complex for small laboratory animals, offering great discrepancy between rats. In veterinary and laboratory studies, the 1st BL line is the longitudinal line of the thoracic costal tubercula [26] or of one-twentieth of the circumference of the thorax [4]. In canine acupuncture, as suggested by Kim et al. in 2004, the location of the 2nd BL line is the longitudinal line extending from the end of the atlas wing, and the 1st BL line is the longitudinal line extending from the middle point between the 2nd BL line and the midline of the back [24]. This method was the simplest and most accurate one to search for the BL lines, and its use resulted in little discrepancy because the atlas wing is a more easily accessible landmark. Therefore, to select the BL acupoints, we used transpositional acupoints based on Kim's method for canine acupuncture of [24]. 

Existing acupoint discrimination instruments use a DC or a high-voltage impulse as an acupoint stimulus source to find a low-impedance point (LIP). These stimulus sources cause cell polarization and electrical shock. Also, when the skin is dry or the skin impedance is high, it is difficult to discriminate acupoints and nonacupoints. At this time, many problems exist because these instruments use high voltage/current and strongly press an electrode probe to discriminate LIPs, which are the reasons for the low reproducibility and discrimination rate of these instruments. To overcome these problems, a new LIP location discrimination system was developed in 1998 by a Korean [27, 28]. This system, the SPAC, uses optimal parameters for the meridian acupoint stimulus. This system uses a 2-electrode method in which a common electrode is fixed at a part of body, after which a measuring probe is contacted on a LIP or a non-LIP. The SPAC system uses 1.28-V (less than the hydrogen ionization voltage [1.36 V]), 4-kHz bidirectional alternating signal with a rectangular waveform (zero crossing start at 6 ms), and 1~20 *μ*A (restricted maximum current, which depends on the impedance of the human body between the two electrodes). Therefore, this method is able to minimize the influence of the body, improve reproducibility, and reduce the pressure of the electrode [27, 29]. Also, because the instrument has a new algorithm to automatically detect of the condition of the skin (wet or dry), it is able to discriminate LIPs by using a low electrode pressure and a low current source. The instrument uses various methods to display the current measured at the LIP: a microampere meter, a level meter, speaker, sound, and a computer monitor.

Several external factors, such as the stratum corneum of the skin, skin moisture, and the characteristics of the electrode, such as electrode polarization and electrode pressure, affect the electrodermal measurements [10, 22, 23]. In particular, the stratum corneum can act as the greatest contributor to the resistance to electrical currents, and skin moisture can affect the electrical conductivity. Therefore, skin integrity and hydration should be maintained during measurements; scratching of the skin should be avoided, and consistent, uniform conditions should be maintained across tests [22]. To decrease the errors due to the differences in skin conditions, in this study, we kept relevant factors constant during the entire procedure. The temperature (23°C) and the humidity (60%) were kept steady during the experiment to maintain skin hydration, and depilation, instead of shaving, was used to remove hair completely from the back because depilation does not damage the skin as much as shaving. Also, to prevent errors due to the differences in the characteristics of the electrode, we performed the measurements under the same conditions for all rats: the same system, one inspector, and no difference in electrode pressure. In particular, we used an electrode guide pole and the weight of a 150-g sinker to reduce the difference in electrode pressure because manual operation might not put a fixed pressure on a electrode, so the results might have lacked reproducibility. An electrode was placed in the electrode guide pole and was linked to the weight. Therefore, the electrode was pressed regularly by the weight and put pressure on the skin vertically.

The BL acupoints on the back showed significantly higher voltages than the reference points. In this SPAC system, a higher voltage indicates a lower electrical resistance and a higher electric current. Furthermore, the acupoints on the 1st BL line showed significantly higher voltage than those on the 2nd BL line. These results coincide with the established idea of Oriental medicine that an important meridian exists on the back, the so-called BL meridian, which has potent acupoints, organ-associated points, managing the functions of internal organs. Of the two BL meridian, the 1st line of the BL meridian is generally known to be the most effective meridian while the 2nd line of the BL meridian acts additionally to assist the function of the 1st BL meridian. 

To identify a specific acupoint showing electrical characteristics, we investigated the difference in the mean voltages between a specific acupoint and the corresponding reference point. Rt BL 16, which showed the biggest difference, is called *Du-Shu*, and it is the governing vessel (GV) meridian association point that is mainly used to control heart disease [24, 26]. Other points with big differences were related to specific organs or had specific functions; Rt BL 27 is associated with the small intestine, and Lt BL 17 is associated with the hematologic function [24, 26]. Therefore, those acupoints are of utility first for investigating the effect of acupuncture related to specific organs or functions. Lt EBL-2 also showed a big difference even though it is not an established acupoint. We think it could be a unique acupoint in rats. Therefore, further studies are needed to investigate the specific role of this point. 

Another well-known characteristic of acupoints is the high density of nerve endings at the acupoints [5, 30–33]. Due to enriched nerve endings, needle stimulation of the nerves in a single acupoint could lead to activation of multireceptors within an acupoint; further, it could propagate and enhance the activation of other acupoints along the same meridian [5, 34, 35]. This result was investigated along the BL and the spleen meridians for which a high density of nerve endings is in perfect accord with the routes of those meridians at the lateral and the medial edges of the paw of a rat, and the outcome was demonstrated at muscles of the proximal portion of the anterior tibialis and of the proximal and the middle-distal portion of the rectus femoris muscle [5]. In this study, several acupoints along the BL meridian on the back were histologically investigated to confirm the nerve densities on the acupoints, and the outcomes were similar to those of the previous studies in that the nerve densities on the acupoints seemed to be higher than those on the reference points. Interestingly, the nerve density at an acupoint seemed to be related to the intensity of the electrical characteristic at that acupoint, with an acupoint exhibiting a higher voltage also exhibiting a higher nerve density. 

Based on our results, transpositional BL acupoints on the back in rats present specific properties as acupoints and meridians, including electrical and histological characteristics. Therefore, those acupoints can be approved more objectively and can beutilized in laboratory animals to confirm the efficacy of acupuncture on the BL meridian. Furthermore, these results support the need for further study to discover the functions of organ-associated points on the BL meridian, as noted in the concept of Oriental medicine, provided the relation between the specific BL acupoint exhibiting electrical and histological characteristics and the specific organ can be identified. 

## Figures and Tables

**Table 1:** Nomenclature of the BL acupoints in this study.

Left side		*Land-	Right side	
^#^2nd BL	^§^1st BL	mark vertebra	^§^1st BL	^#^2nd BL
EBL-1	BL11	T1	BL11	EBL-1
BL41	BL12	T2	BL12	BL41
BL42	BL13	T3	BL13	BL42
BL43	BL14	T4	BL14	BL43
BL44	BL15	T5	BL15	BL44
BL45	BL16	T6	BL16	BL45
BL46	BL17	T7	BL17	BL46
^†^EBL-3	^†^EBL-2	T8	^†^EBL-2	^†^EBL-3
^†^EBL-5	^†^EBL-4	T9	^†^EBL-4	^†^EBL-5
BL47	BL18	T10	BL18	BL47
BL48	BL19	T11	BL19	BL48
BL49	BL20	T12	BL20	BL49
BL50	BL21	T13	BL21	BL50
BL51	BL22	L1	BL22	BL51
BL52	BL23	L2	BL23	BL52
^†^EBL-6	BL24	L3	BL24	^†^EBL-6
^†^EBL-7	BL25	L4	BL25	^†^EBL-7
^†^EBL-8	BL26	L5	BL26	^†^EBL-8
BL53	BL27	L6	BL27	BL53

*T: thoracic vertebra, L: lumbar vertebra, individual BL acupoint located on the 1st BL or the 2nd BL line to the lower border of the spinous process of each landmark vertebra.

^§^1st BL: 1st urinary bladder meridian (the longitudinal line extending from the middle point between the 2nd BL line and the midline of the back), ^#^2nd BL: 2nd urinary bladder meridian (the longitudinal line extending from the end of the atlas wing), ^†^EBL: extra BL acupoints, which are acupoints that have not been previously established.
